# The Polypyrimidine Tract-Binding Protein Is a Transacting Factor for the Dengue Virus Internal Ribosome Entry Site

**DOI:** 10.3390/v16111757

**Published:** 2024-11-09

**Authors:** Leandro Fernández-García, Jenniffer Angulo, Marcelo López-Lastra

**Affiliations:** 1 Laboratorio de Virología Molecular, Instituto Milenio de Inmunología e Inmunoterapia, Departamento de Enfermedades Infecciosas e Inmunología Pediátrica, Centro de Investigaciones Médicas, Escuela de Medicina, Pontificia Universidad Católica de Chile, Marcoleta 391, Santiago 8330024, Chile; leandrofg1990@gmail.com (L.F.-G.); jangulc@uc.cl (J.A.); 2 Facultad de Ciencias Biológicas, Pontificia Universidad Católica de Chile, Avenida Libertador Bernardo O’Higgins 340, Santiago 8331150, Chile

**Keywords:** dengue virus, IRES, translation initiation, PTB, ITAFs

## Abstract

*Dengue virus* (DENV) is an enveloped, positive sense, single-stranded RNA virus belonging to the *Flaviviridae*. Translation initiation of the DENV mRNA (vRNA) can occur following a cap-dependent, 5′-3’end-dependent internal ribosome entry site (IRES)-independent or IRES-dependent mechanism. This study evaluated the activity of DENV IRES in BHK-21 cells and the role of the polypyrimidine-tract binding protein (PTB) isoforms PTB1, PTB2, and PTB4 as IRES-transacting factors (ITAFs) for the DENV IRES. The results show that DENV-IRES activity is stimulated in DENV-replicating BHK-21 cells and cells expressing the *Foot-and-mouth disease virus* leader or *Human rhinovirus* 2A proteases. Protease activity was necessary, although a complete shutdown of cap-dependent translation initiation was not a requirement to stimulate DENV IRES activity. Regarding PTB, the results show that PTB1 > PTB2 > PTB4 stimulates DENV-IRES activity in BHK-21 cells. Mutations in the PTB RNA recognition motifs (RRMs), RRM1/RRM2 or RRM3/RRM4, differentially impact PTB1, PTB2, and PTB4’s ability to promote DENV IRES-mediated translation initiation in BHK-21 cells. PTB1-induced DENV-IRES stimulation is rescinded when RRM1/RRM2 or RRM3/RRM4 are disrupted. Mutations in RRM1/RRM2 or RRM3/RRM4 do not affect the ITAF activity of PTB2. Mutating RRM3/RRM4, but not RRM1/RRM2, abolishes the ability of PTB4 to stimulate the DENV IRES. Thus, PTB1, PTB2, and PTB4 are ITAFs for the DENV IRES.

## 1. Introduction

*Dengue virus* (DENV) is an enveloped, positive-sense, single-stranded RNA virus that, in humans, can cause diseases ranging from self-limiting dengue fever to life-threatening dengue hemorrhagic fever [1]. Upon cell entry, the 5′capped(m^7^GpppA_2′OMe_) DENV genomic RNA directly acts as the viral messenger RNA (vRNA) [2,3]. Translation initiation of the DENV vRNA can be cap-dependent or cap-independent [2,3,4,5,6,7]. Two cap-independent initiation mechanisms have been reported for the DENV vRNA [5,6], neither of which has been fully characterized. One is a 5′-3′end-dependent internal ribosome entry site (IRES)-independent initiation mechanism [5], and the other is an IRES-mediated initiation mechanism [6,7]. This report focuses on the DENV IRES, present within the 5′untranslated region (UTR) of the vRNA [6].

In cap-dependent translation initiation (reviewed in detail in [8,9]), the eukaryotic initiation factor (eIF) 4F, constituted by the cap-binding protein eIF4E, an ATP-dependent RNA helicase eIF4A, and a scaffold protein eIF4G, recognizes the cap structure at the 5′end of the mRNA and bridges it with the 40S ribosomal subunit. The 40S ribosomal subunit is recruited to the mRNA as part of the 43S preinitiation complex composed of the subunit bound to eIF2-GTP/Met-tRNA_i_ (ternary complex), eIF1A, eIF1, eIF3, and eIF5 [8,9], via the eIF4G-eIF3 interaction. The recruited 43S complex scans the 5′UTR of the mRNA in a 5′ to 3′ direction until the initiation codon is encountered, leading to 60S subunit joining and assembly of an elongation-competent 80S ribosome [8,9].

In general, an IRES corresponds to an RNA element, which allows assembly of the translational machinery at a position close to or directly at the initiation codon, independently of the 5′end of the mRNA [10,11]. For many viral IRESs, RNA-binding proteins (RBP), referred to as IRES-transacting factors (ITAFs), regulate IRES function [10,12]. The molecular mechanisms driving 40S ribosomal subunit recruitment to the DENV IRES and ITAFs that might participate in the process remain unknown.

In this study, we sought to further characterize the DENV vRNA IRES-dependent translation initiation. Experiments were conducted in baby hamster kidney fibroblast cells (BHK-21), selected because they support DENV replication and cap-independent translation initiation from the DENV vRNA [5,6,7,13,14]. Our results show that inhibiting cap-dependent translation by replacing the 5′cap with a nonfunctional cap-analog is insufficient to enhance DENV cap-independent translation initiation in a monocistronic virus-like mRNA context. However, cap-independent translation of the DENV mRNA is increased in BHK-21 cells expressing the *Foot-and-mouth disease virus* leader protease (L^pro^). Next, we provide data showing that DENV IRES activity is stimulated in BHK-21 cells replicating DENV or expressing the *Human Rhinovirus* 2A proteases (2A^pro^). We also show that the three isoforms of the polypyrimidine tract-binding protein (PTB1, PTB2, and PTB4) are ITAFs that stimulate DENV IRES activity (PTB1 > PTB2 > PTB4) when overexpressed in BHK-21 cells. Finally, we provide evidence showing that mutations in the PTB RNA recognition motifs (RRMs) differentially impact PTB1, PTB2, and PTB4’s ability to promote DENV IRES-mediated translation initiation in BHK-21 cells.

## 2. Materials and Methods

### 2.1. Plasmids

The plasmids pGL5′3′DV (kindly provided by Dr. A. Gamarnik, Fundación Instituto Leloir, Argentina), dl ∆EMCV, dl DENV IRES, and dl HCV IRES have been described previously [7,15,16,17,18]. The monocistronic version of the DENV-FLuc mRNA reporter was constructed from pGL5′3′DV as described in [7]. The pCI-Neo-HA (pRLuc) and Glo-FLuc (Globin) vectors (kindly provided by Dr. R. Soto-Rifo, ICBM, Universidad de Chile, Chile) were described in [19,20,21,22]. Plasmids expressing either the functional wild-type (2A^pro^-WT) or an inactive mutant (2A^pro^-Mut) of human Rhinovirus 2A protease (genotype A16) were kindly supplied by Drs. A.C. Palmenberg and K.Watters (University of Wisconsin, Madison, WI, USA) and validated in [19]. The foot-and-mouth disease virus L protease-expressing plasmid was generously provided by Dr. G. Belsham (Department of Veterinary and Animal Sciences, University of Copenhagen, Denmark) and described in [23]. The encoding plasmid for DENV 2 genome pDVWS601, described in [24], was kindly provided by Dr. Ali Amara (INSERM, Institut Jean Bernard, Hôpital Saint-Louis, Paris, France). The PTBs-tagged expressing plasmids (pcDNA4/HisMax-PTB1, pcDNA4/HisMax-PTB2, pcDNA4/HisMax-PTB4, pCINeo-PTB2-FLAG, pCINeo-PTB4-FLAG, pcDNA4/HisMax-PTB1m1.2, pcDNA4/HisMax-PTB1m3.4, pCINeo-PTB4m1.2-FLAG, and pCINeo-PTB4m3.4-FLAG) were described in [17,25,26]. The pCINeo-PTB2m1.2-FLAG and pCINeo-PTB2m3.4-FLAG vectors were constructed by overlapping PCR from PTB4 mutant vectors as described in [25]. Briefly, primers *Bam*HI-PTB-F (5′-GGATCCATGGACGGCATTGTCCCAG-3′) and PTB2N-terROv (5′-TACGGAGAGGCGAAGGCCGCGGCCATG-3′) were used to generate the N-terminal segment and primers *Eco*RI-PTB-R (5′-GAATTCCTAGATGGTGGACTTGGAG-3′) and PTB2C-terFO (5′-GCGGCCTTCGCCTCTCCGTATGCAGG-3′) for the C-terminal segment. The commercial plasmid, pSP64 Poly(A) (#P1241, Promega Corporation, Madison, WI, USA) was used as irrelevant DNA to standardize the total DNA amounts transfected in each condition. The pcDNA3.1-LacZ, encoding the β-galactosidase, was used to control the DNA transfection efficiency, as previously described [17,25]. All constructs used in this study were confirmed by sequence analysis (Psomagen Inc., Rockville, MD, USA).

### 2.2. Cell Culture 

BHK-21 (ATCC CCL-10) cells were grown in Dulbecco’s modified Eagle’s medium (DMEM; #SH30022, HyClone, GE Healthcare Life Sciences, Marlborough, MA, USA) containing 10% fetal bovine serum (#SH30910, Hyclone, GE Healthcare Life Sciences), 1% penicillin-streptomycin (1000 U/ mL) (#SV30010, Hyclone, GE Healthcare Life Sciences), and 1% amphotericin B (25 mg/mL) (#SV30078.01, Hyclone, GE Healthcare Life Sciences) at 37 °C in a 5% CO_2_ atmosphere.

### 2.3. DNA Transfection Assays 

For cotransfection assays, the BHK-21 cells were seeded at 6 × 10^4^ cells per well in a 24-well culture plate 24 h before transfection. DNA cotransfections were performed over cells at a 60–70% confluency. For the 2A^pro^ experiments, 200 ng of pRLuc vector or 200 ng of dl plasmid (dl ∆EMCV, dl HCV IRES, dl DENV IRES, dl EMCV IRES) were cotransfected with 500 ng of 2A^pro^-WT or 2A^pro^-Mut using polyethyleneimine (PEI; #23966 Gibco BRL, Life Technologies Corporation, Carlsbad, CA, USA). For the PTB overexpression experiments, 200 ng of dl DENV IRES together with increasing amounts of plasmid pcDNA4/HisMax-PTB1, pcDNA4/HisMax-PTB2, and pcDNA4/HisMax-PTB4 were transfected with PEI. For experiments using the mutant-PTBs, 200 ng of the dl DENV IRES was cotransfected with 1000 ng of the PTBs expression vectors. The pSP64 Poly(A) plasmid was used as a filling DNA to keep the total amounts constant in all conditions. In all experiments, 24 h after transfection, the culture medium was removed, and the cells were harvested using Passive Lysis buffer supplied with the DLR^TM^ Assay System (#E1910, Promega Corporation) according to the manufacturer’s protocols. The lysed cells were kept for measurement of firefly luciferase (FLuc) and *Renilla* luciferase (RLuc) activity and Western blotting (see below).

### 2.4. In Vitro Transcription 

Capped monocistronic RNAs were transcribed using T7 RNA polymerase (mMESSAGE mMACHINE Kit, #AM1344, Ambion, Thermo Fisher Scientific, Waltham, MA, USA). Plasmids pGL5′3′DV and FMDV-L protease-expressing vector were linearized with *XbaI* (#ER0681, Thermo Fisher Scientific) and the Glo-FLuc plasmid with *EcoRI* (#ER0271, Thermo Fisher Scientific), respectively, as was described in [7]. Acapped-RNA was also synthesized using the mMESSAGE mMACHINE Kit (Ambion), replacing the 2X T7 NTP/CAP mix with a homemade 2X T7 NTP/ACAP mix. The 2X T7 NTP/ACAP mix contained the A(5′)ppp(5′)A-cap analog (#NU-506-5, Jena Bioscience, Jena, Thuringia, Germany) and NTPs (7.5 mM final concentration of CTP, ATP, UTP, and 1.5 mM final concentration of GTP; #R0471, #R0461, #R0451, #R0441, Thermo Fisher Scientific Inc.). The transcription reactions were incubated for two hours at 37 °C. To generate DENV2 gRNA, pDVWS601 was digested with *XbaI* (Thermo Fisher Scientific), and 1 µg of the linearized plasmid was used as a template using the mMESSAGE mMACHINE Kit (Ambion); however, the transcription reaction was incubated overnight at room temperature followed by 2 h at 37 °C. When indicated, a poly(A) tail was added after mRNA synthesis using the Poly(A) tailing Kit (#AM1350, Thermo Fisher Scientific) following the manufacturer’s instructions. All synthesized mRNAs were treated with 2 U of Turbo DNase for 30 min at 37 °C, precipitated for 1 h at −20 °C in the presence of 2.5 M LiCl, centrifuged at 16,000× *g* for 20 min at 4 °C, washed twice with 70% ethanol, resuspended in 50 µL of nuclease-free water, and purified through G50 columns [27]. RNA concentrations were determined spectrophotometrically (NanoPhotometer N60, Implen, Westlake Village, CA, USA), and the RNA integrity was monitored by electrophoresis on agarose gels.

### 2.5. RNA Transfection

For RNA transfection experiments, 3 × 10^4^ BHK-21 cells per well were seeded in 48-well plates. After 24 h and at 80–90% of confluency, the cells were transfected with 0.15 pmol of each in vitro transcribed mRNA (pRLuc, Globin, Acap-Globin, DENV-FLuc, Acap-DENV-FLuc, FMDV L) using a Lipofectamine 2000 system. At 6 h.p.t., the culture medium was removed, and the cells were harvested using Passive Lysis buffer supplied with the DLR^TM^ Assay System (Promega), as described above. Cell lysis was used to measure FLuc activity and for Western blotting (see below).

### 2.6. DENV2 gRNA and Time Course Experiments

The BHK-21 cells were seeded at 5 × 10^4^ cells per well in a 24-well plate, and 24h later, the cells were transfected with 200 ng of dl DENV IRES or dl ∆EMCV together with 10 ng of βgal encoding DNA using PEI. Then, 4 h.p.t., the culture medium was replaced with DMEM 1% FBS and transfected or not with 2 µL (1X) or 10 µL (5X) of DENV gRNA transcription reaction using the Lipofectamine 2000 system (#11668-019; Thermo Fisher Scientific). The cells were harvested at 24 and 48 h.p.t. using the Passive Lysis buffer supplied with the DLR^TM^ Assay System. Samples were kept for the measurement of luciferase activities and for DENV RNA quantification (see below).

### 2.7. RNA Extraction and DENV RT-qPCR

For quantifying the amount of monocistronic mRNA in cells upon transfection in each condition (Globin, DENV-FLuc, Acap-Globin, Acap-DENV-FLuc), cells were lysed at the indicated time with Passive Lysis buffer supplied with the DLR^TM^ Assay System and total RNA was recovered using the QIAmp^®^ Viral RNA Mini Kit (# 52906, QIAGEN, Germany) following the manufacturer’s instructions. Total RNA was eluted in nuclease-free water (100 µL), and RNA concentrations were determined spectrophotometrically (NanoPhotometer N60, Implen, Westlake Village, CA, USA). All RNA samples were diluted in nuclease-free water aliquots of 25 ng/µL to be quantified using the absolute quantification method through Real-time RT-qPCR. The Real-time RT-qPCR experiments were carried out using the Brilliant II SYBR Green RT-qPCR one Step Master Mix (#600835, Agilent Technologies, Santa Clara, CA, USA) in a CFX96 Touch Real-Time PCR Detection system (BioRad, Hercules, CA, USA). For this purpose, a standard curve (5 ng to 5 × 10^−7^ ng) was constructed using the in vitro transcribed monocistronic mRNAs (Globin, DENV-FLuc). The FLuc coding region was detected with firefly sense (5′-ACTTCGAAATGTCCGTTCGG-3′) and firefly antisense (5′-GCAACTCCGATAAATAACGCG-3′) primers, as previously described [19,28,29]. All samples were run in duplicate, and the Cp (Crossing point) average value was used to interpolate the RNA concentration (ng) within the standard curve. The standard curves were accepted when slopes were between 3.5 and 3.0 and the correlation coefficients were all >0.990.

For viral DENV RNA quantification, the cells were lysed at 0, 24, and 48 h.p.t. with Passive Lysis buffer supplied with the DLR^TM^ Assay System and subjected to the same procedure for the RNA extraction method mentioned above. The Real-time RT-qPCR was carried out in a CFX96 Touch Real-Time PCR Detection system following all the recommendations of the LightMix Kit Dengue Virus EC (#40-0439-32, TIB Molbiol, Berlin, Germany), which can identify all four dengue serotypes. Each Cp value for DENV RNA was normalized relative to the Cp value obtained for Ribosomal 18S rRNA, used as a reference gene, through the 2^−ΔΔCt^ method as previously described [30,31].

### 2.8. Immunofluorescence (IFI) and Confocal Microscopy

BHK-21 cells were seeded at 5 × 10^4^ cells per well in 12 mm coverslips in a 24-well plate. Then, 24 h later, the culture medium was changed and replaced with DMEM 1% FBS and transfected or not transfected with 2 µL of DENV gRNA using the Lipofectamine 2000 system as described above. At 48 h.p.t., cells were washed with PBS and fixed with 4% paraformaldehyde for 20 min at room temperature. Cells were permeabilized using 0.025% Saponin in a blocking solution (1% BSA) for 1 h at 37 °C. DENV2 NS3 was detected using the primary anti-DENV NS3 antibody (#PA5-32199, Thermo Fisher Scientific) used at 1:500 dilution in blocking solution overnight at 4 °C, and the detection of endogenous PTB was performed using the anti-PTBP2 (A-10) antibody (#sc-376316, Santa Cruz Biotechnology, Dallas, TX, USA) at a 1:250 dilution. Cells were washed 3 times with PBS for 5 min at room temperature and incubated with the secondary anti-rabbit-AlexaFluor 488 (#A32723, Thermo Fisher Scientific) or anti-mouse Alexa Fluor 555 (#A21422) used at 1:500 dilution in blocking solution for 1 h at 37 °C. The cells were rewashed as described above, and coverslips were mounted with VECTASHIELD^®^ HardSet™ Antifade Mounting Medium with 4′,6-diamidino-2-phenylindole (DAPI) (#H-1500-10, Vector Laboratories, Inc., Burlingame, CA, USA). For the infection experiments, Dengue virus type 2 strain New Guinea C (ATCC; VR-1584) was propagated in BHK-21 cells cultured in DMEM 2% FBS 5% CO_2_ for seven days. The virus stocks’ 50% tissue culture infectious dose (TCID_50_) was determined by the endpoint cytopathic effect at five days post-infection in BHK-21 maintained in DMEM 2% FBS 5% CO_2_. Virus stocks were stored at −80 °C until needed. For endogenous PTB localization in cells replicating DENV, BHK-21 cells were seeded at 5 × 10^4^ cells per well in 12 mm coverslips in a 24-well plate, and 24 h later, the cells were infected with DENV2 NGC at 1 TCID_50_. After 48 h.p.i., cells were washed with PBS and fixed with 4% paraformaldehyde for 20 min at room temperature. The DENV-NS3 protein was detected, as indicated above. Images were captured and analyzed by confocal laser microscopy (Nikon C2+, Melville, NY, USA) using immersion oil and a 60X objective. Images were captured independently by multitracking imaging of each channel to eliminate possible crosstalk between fluorochromes. Images were analyzed using ImageJ 1.38 software (Windows version of NIH Image http://rsb.info.nih.gov/nih-image/ (accessed on 3 November 2024)) as in [32].

### 2.9. Fluorescence Microscopy

BHK-21 cells were seeded at 5 × 10^4^ cells per well in 12 mm coverslips in a 24-well plate. Twenty-four hours later, the cells were transfected with 500 ng of His-PTB1, His-PTB2, His-PTB4, or pSP64 Poly(A) vector plus 200 ng of dl DENV IRES using PEI (#23966 Gibco BRL, Life Technologies Corporation). After 24 h.p.t., the cells were washed with PBS and fixed with 4% paraformaldehyde for 20 min at room temperature. Permeabilization was performed using PBS-Triton X-100 (0.03%) in a blocking solution (10% BSA) for 1 h at 37 °C. The detection of His-PTBs was performed using the monoclonal anti-polyhistidine antibody (#H1029, Sigma-Aldrich, St. Louis, MO, USA) at a 1:500 dilution. Cells were washed 3 times with PBS for 5 min at room temperature and incubated with the secondary antibody anti-mouse Alexa Fluor 555 (#A21422) used at 1:500 dilution in blocking solution for 1 h at 37 °C. The cells were rewashed as described above, and coverslips were mounted with VECTASHIELD^®^ HardSet™ Antifade Mounting Medium with 4′,6-diamidino-2-phenylindole (DAPI) (#H-1500-10, Vector Laboratories, Inc., Burlingame, CA, USA). Images were captured and analyzed in an Olympus BX51 Microscope (Center Valley, PA, USA) using a PlanApo N 60x/1.42na oil objective. Images were captured and analyzed using ImageJ 1.38 software.

### 2.10. siRNA-DNA Cotransfection

BHK-21 cells were seeded at 5 × 10_4_ cells per well in a 24-well culture plate. At a confluence of 70–80%, cells were transfected with a mixture of anti-PTB siRNAs (50 nM of each, 150 nM in total): siRNA PTBP1; 5′-AACUUCCAUCAUUCCAGAGAA-3′, siRNA PTBP2;5´-GAGAGGAUCUGACGAACUA-3′, and SMART pool siGENOME PTBP1 siRNA (# M-003528-02, GE Healthcare Dharmacon Inc., Piscataway, NJ, USA) as described in [17,25], together with 200 ng of dl DENV IRES and 375 ng of pSP64 Poly(A) plasmids using Lipofectamine 2000 system. As a control, 150 nM of Silencer Select Negative Control N°1 siRNA (#4404021, Thermo Fisher Scientific) was used (herein referred to as the scramble RNA, scRNA). After 48 h of siRNA and dl DENV IRES transfection, the cells were harvested, and the RLuc and FLuc activities were measured.

### 2.11. Luciferase and ß-Galactosidase Activity Measurement

Cell lysates (in passive buffer) were used for the measurement of FLuc and Rluc activity using the Dual-Luciferase^®^ Reporter Assay System (#E1960, Promega corporation) and the ß-gal activity with the Beta-Glo^TM^ Assay System (#E4720, Promega Corporation) according to the manufacturer’s instructions on a Sirius L Tube Luminometer (Titertek-Berthold Detection Systems GmbH, Pforzheim, Germany). Data are expressed as Relative Luciferase Units (RLU), Relative Luciferase Activity (RLA (%)), or Relative Translation Activity (RTA), which correspond to the FLuc/RLuc ratio, an index of the IRES activity [7,17,19].

### 2.12. Surface Sensing of Translation (SUnSET)

The impact of 2A^pro^-WT and 2A^pro^-Mut on global cellular protein synthesis was assessed using the SUnSET technique, as outlined previously [33,34]. BHK-21 cells were seeded at a density of 6x10^4^ cells per well in a 24-well plate one day before transfection. The cells were transfected with 500 ng of 2A^pro^-WT or 2A^pro^-Mut vectors using PEI. After 48 h, cells underwent a 10 min puromycin pulse (1 μg/mL). As a translation inhibition control, non-transfected cells were pre-treated with dithiothreitol (DTT, 2.5 mM) for 90 min before the puromycin pulse. Western blot analysis was employed to detect de novo synthesized proteins containing puromycin, as indicated below.

### 2.13. Western Blotting

Protein content was determined using the Bio-Rad protein assay (#500-0006, Bio-Rad Laboratories, Inc., Hercules, CA, USA). For endogenous PTB detection in siRNA experiments and recombinant PTB detection, 70 µg and 40 µg of total protein were used, respectively. All these lysates were resolved and transferred for 1 h at 100 V to a 0.45 µM nitrocellulose membrane (#10600002, Amersham, GE Healthcare, Life Sciences). For SUnSET experiments and proteolytic cleavage of eIF4G after the treatment with 2A^pro^, 40 µg of total protein was resolved by sodium dodecyl sulfate-polyacrylamide gel electrophoresis (SDS/PAGE) on a 10% gel and transferred for 1.5 h at 100 V to a 0.45 µM nitrocellulose membrane. All membranes were blocked with TBS containing 5% skimmed milk and 0.1% Tween 20 for 1 h at room temperature, washed three times with TBS containing 0.1% Tween 20, and incubated overnight with the primary antibody. Monoclonal anti-polyhistidine antibody (#H1029, Sigma-Aldrich, St. Louis, MO, USA) at a 1:5000 dilution, anti- Flag antibody (#F3165, Sigma-Aldrich) at a 1:500 dilution, anti-GAPDH antibody (#MAS-15738, Thermo Scientific) at a 1:5000 dilution, anti-PTBP-2 (A-10) antibody (#sc-376316, Santa Cruz Biotechnology, Dallas, TX, USA) at a 1:1000 dilution, polyclonal anti-eIF4G antibody (H-300) (sc-11373, Santa Cruz Biotechnology) at a 1:1000 dilution, or anti-puromycin antibody (clone 12D10) (#MABE343, EMD Millipore, Temecula, CA, USA) at a 1:15,000 dilution were used as the primary antibody. An anti-mouse IgG- HRP conjugate (#074-1806; KPL Inc., Gaithersburg, MD, USA) or anti-rabbit IgG-HRP conjugate (#12-348, Sigma-Aldrich) was used as a secondary antibody at a 1:10,000 or 1:5000 dilution, respectively. The expression of the recombinant protein was visualized by enhanced luminescence using 4-hydroxycinnamic acid (#800237, Merck Millipore, Burlington, MA, USA) and luminol (#09253, Fluka, Sigma-Aldrich) or SuperSignal^TM^ West Femto-ECL (#34096, Thermo Scientific). The Western blot films (Fuji medical X-ray film Super HR-U 30 or Hyblot CL (Cat: DV-3012, Denville Scientific Inc., New York, Metuchen, NJ, USA)) were digitized using a CanonScan 9950F scanner, or membrane chemiluminescence was captured using an Alliance 2.7 imaging system (UVItec Cambridge, Topac Inc., 231 CJC Highway, Cohasset, MA, USA). For semiquantitative protein analysis, optical densities (ODs) were determined using the ImageJ 1.38 software.

### 2.14. Statistical Analysis and Sequence Analysis

All the statistical data analysis and graphics were performed using the GraphPad Prism v10.2.1 for Windows (GraphPad Software, Boston, MA, USA, www.graphpad.com). Serial Cloner 2.6.1 program was used for sequence alignments and analysis.

## 3. Results

### 3.1. DENV Cap-Independent Translation Initiation Is Stimulated in BHK-21 Cells Expressing the FMDV L Protease

**Figure 1:**
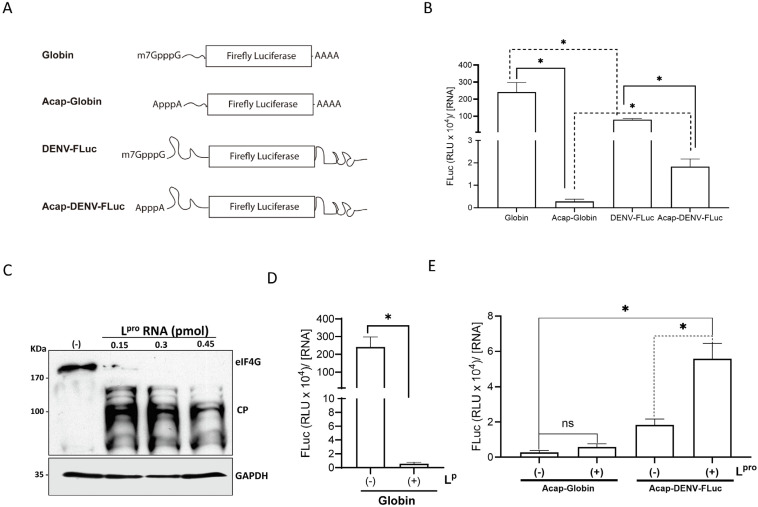
**DENV 5′UTR in the context of monocistronic mRNAs has cap-independent translation activity in BHK-21 cells**. (**A**) Schematic representation of the in vitro-transcribed RNAs used in the studies. The capped (m^7^GpppG-) and A-capped (ApppA) DENV-FLuc RNA (virus-like) were transcribed from the pGL5′3′DV plasmid [15]. The DENV-FLuc RNA comprises the 5′UTR-C62 (nt 1 to 160) and the 3′UTR of the DENV 2 (strain 16681; GeneBank NC_001474.2) mRNA flanking the firefly luciferase (FLuc) open reading frame (ORF) [7]. Capped or A-capped polyadenylated globin RNA reporters that harbor the 5′UTR of the globin mRNA and a poly(A) tail flanking the FLuc ORF were also used [7]. (**B**) BHK-21 cells were transfected with the indicated RNA. Total RNA was recovered, and the RNA levels were determined by real-time RT-qPCR. Cell lysates were used to determine the FLuc activity. Data are presented as relative luminescent units (RLUs) normalized to the relative FLuc RNA abundance. Statistical analysis was performed using one-way ANOVA, followed by Tukey’s multiple comparison test. Values are the mean (±SEM) for three independent experiments, each performed in duplicate (* *p* < 0.05). (**C**) In vitro transcribed RNA encoding for the FMDV L protease (0.15, 0.3, or 0.45 pmol) was transfected or not (-) in BHK-21 cells. Western analysis was performed using polyclonal antibodies against eIF4G [35]. Positions of molecular mass standards (in kDa) are shown. The eIF4G cleavage product (CP) has been previously characterized [35]. (**D**) The Globin RNA was cotransfected with an irrelevant RNA or in vitro transcribed RNA encoding for the FMDV L protease (0.15 pmol). Real-time RT-qPCR, determined RNA levels, and cell lysates were used to determine the FLuc activity described above. Data are presented as RLU normalized to the relative FLuc RNA abundance. Statistical analysis was performed using a *t*-test (* *p* < 0.05). Values are the mean (±SEM) for three independent experiments, each performed in duplicate. (**E**) The A-cap-Globin or Acap-DENV RNA was transfected with an irrelevant RNA or in vitro transcribed RNA encoding for the FMDV L protease (0.15 pmol). Total RNA was recovered, and the RNA levels were determined by real-time RT-qPCR. In parallel, cell lysates were used to determine the FLuc activity. Data are presented as RLU normalized to the relative FLuc RNA abundance. Statistical analysis was performed using one-way ANOVA, followed by Tukey’s multiple comparison test. Values are the mean (±SEM) for three independent experiments, each performed in duplicate. * *p* < 0.05, and ns, not significant.

The DENV vRNA is expected to initiate translation using a cap-dependent or a cap-independent mechanism [5,6]. However, DENV cap-independent translation is low but increases when cap-dependent translation initiation is inhibited [5]. To further these observations, in vitro transcribed monocistronic Globin m^7^GpppG-3′Poly(A) (Globin RNA), Globin ApppA-3′Poly(A) (Acap-Globin RNA), virus-like mRNAs DENVm^7^GpppG-3′UTR (DENV-FLuc RNA), or DENVApppA-3′UTR (Acap-DENV-FLuc RNA) (depicted in Figure 1A) were transfected in BHK-21 cells. Four hours post-transfection (h.p.t.), cells were lysed, luciferase activity was determined, and total RNA was extracted from the cell lysates. Total RNA was used as a template in an RT-qPCR assay to determine the relative amount of FLuc-encoding mRNA in cells, and luciferase activity was normalized to the amount of FLuc-encoding RNA. In cells, replacing the m^7^GpppG-cap for an ApppA-cap abrogated translation from the Globin RNA reporter. In concordance with earlier reports [4,7], exchanging the m^7^GpppG-cap with an ApppA-cap in the DENVm^7^GpppG-3′UTR RNA led to a sharp reduction, not a total inhibition, in translation (Figure 1B), indicating that in BHK-21, translation initiation of these mRNAs is mainly, but not exclusively, cap-dependent.

Next, we evaluated the impact of directly inhibiting cap-dependent translation on the monocistronic mRNAs. For this, different amounts of in vitro synthesized L^pro^ encoding mRNA (0.15, 0.3, or 0.45 pmol) were transfected in BHK-21 cells. The cleavage of eIF4G by the L^pro^ leads to the inhibition of cap-dependent (eIF4E-dependent) but not cap-independent translation initiation [36,37]. The expression of the L^pro^ was indirectly assessed by monitoring the cleavage of eIF4G by Western blot (Figure 1C). Next, BHK-21 cells were transfected with Globin mRNA, Acap-Globin, and Acap-DENV-FLuc, with or without the L^pro^ mRNA (0.15 pmol). In the presence of the L^pro^, cap-dependent translation initiation from the Globin mRNA was abrogated (Figure 1D). As anticipated, FLuc activity of the Acap-Globin mRNA was low, confirming the lack of translation from this control RNA. The presence of the L^pro^ translation from the Acap-DENV-FLuc mRNA was significantly increased (by 311%) in BHK-21 cells (Figure 1E). These results show that the expression of L^pro^ hinders cap-dependent while enhancing DENV cap-independent translation initiation in BHK-21 cells.

### 3.2. DENV IRES Activity Is Stimulated in DENV-Infected Cells

**Figure 2:**
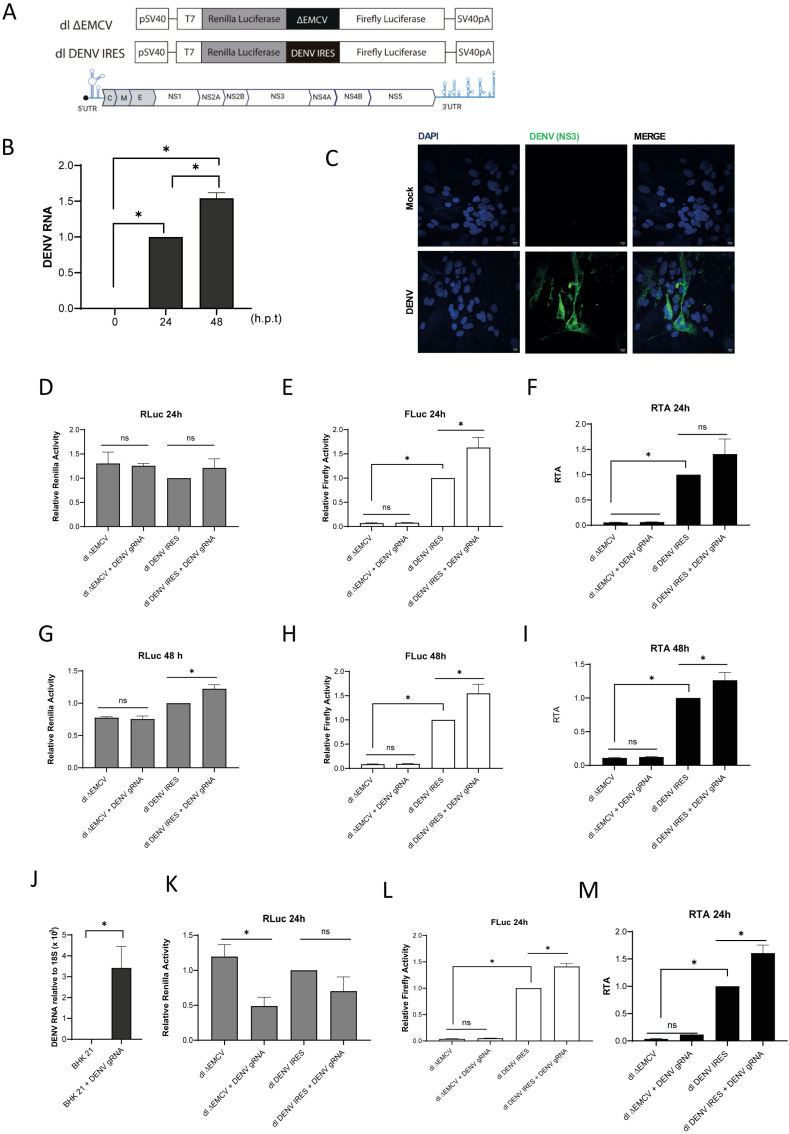
**DENV IRES activity in BHK-21 cells expressing the DENV gRNA.** (**A**) Schematic representation of the dual-luciferase (dl) plasmids and DENV RNA used in the studies. In these dl-plasmids, the first cistron corresponds to the *Renilla* luciferase (RLuc) ORF, while the second cistron corresponds to the FLuc ORF. The dl ∆EMCV that harbors in the intercistronic region a defective encephalomyocarditis virus (∆EMCV) 5′UTR that lacks IRES activity has been previously described [16]. The dl DENV IRES harbors the 5′UTR (nts 1-96; GeneBank U87411) of the DENV 2 (strain 16681) mRNA within the intercistronic region, previously described as DENV IRES [7]. (**B**) dl-vectors indicated were transfected in BHK-21 cells, and at 4 h.p.t., the culture medium was replaced and cells were transfected, or not, with DENV gRNA (1X). DENV RNA in cells was monitored at times 0, 24, and 48 h.p.t. Statistical analysis was performed using one-way ANOVA, followed by Tukey’s multiple comparison test (* *p* < 0.05, and ns, not significant). (**C**) At 48 h.p.t. of the DENV gRNA, the expression of the viral NS3 protein was detected by immunofluorescence (IF) using a commercially available anti-DENV NS3 antibody. Vectashield with DAPI was used as a mounting media. Images were captured and analyzed by confocal laser microscopy (Nikon C2+, Melville, NY, USA) and processed by Image J. Scale bar = 10 μm. (**D**–**I**) RLuc and FLuc activities were determined at 24 (**D**–**F**) and 48 (**G**–**I**) h.p.t. of the DENV gRNA. RLuc (**D**,**G**) and FLuc (**E**,**H**) activities expressed relative to those obtained in cells transfected with the dl DENV IRES plasmid alone arbitrarily set to 1. Results are also expressed as relative translation activity (RTA) relative to the dl DENV IRES activity (**F**,**I**), which was set to 1. (**J**–**M**) dl-vectors indicated were transfected in BHK-21 cells, and at 4 h.p.t., the culture medium was replaced and cells were transfected, or not, with DENV gRNA (5X). (**J**) DENV RNA in cells was monitored at times 0 and 24. Statistical analysis was performed using one-way ANOVA, followed by Tukey’s multiple comparison test (* *p* < 0.05, and ns, not significant). (**K**–**M**) RLuc and FLuc activities were determined at 24 h.p.t. of the DENV gRNA. RLuc (**K**) and FLuc (**L**) activities expressed relative to those obtained in cells transfected with the dl DENV IRES plasmid alone arbitrarily set to 1. (**M**) Results are also expressed as relative translation activity (RTA) relative to the dl DENV IRES activity, which was set to 1. Values are the mean (+/− SEM) for at least three independent experiments, each performed in duplicate. RTA corresponds to the FLuc/RLuc ratio that is used as an index of IRES activity. Statistical analyses were performed using one-way ANOVA and Tukey’s multiple comparison test (* *p* < 0.05, and ns, not significant).

Translation initiation of the DENV vRNA can follow a cap-dependent, a 5′-3′end-dependent IRES-independent [5], and an IRES-dependent mechanism [6,7]. The results presented above (Figure 1) can be due to either cap-independent mechanism. To exclusively focus on the IRES-mediated translation initiation, we switched to a previously reported and well-characterized plasmid encoding for a dual luciferase (dl) bicistronic mRNA [7]. The bicistronic system harbors an upstream *Renilla* luciferase (RLuc) ORF and downstream firefly luciferase (FLuc) ORF dl-RNA, with the 5′UTR of the DENV-2 mRNA (nt 1 to 96 of DENV 2 mRNA; GenBank accession no. NC_001474.2) placed between both cistrons (Figure 2A), the dl DENV IRES [7]. First, we sought to determine if the DENV IRES was active in BHK-21 cells replicating DENV. To this end, BHK-21 cells were transfected with the dl DENV IRES or with the dl ∆EMCV DNA (Figure 2A), which has a deleted 5′UTR of the *Encephalomyocarditis virus* (∆EMCV) RNA in its intercistronic space and lacks IRES activity [16]. Four h.p.t. with the dl-vectors cells were transfected, or not, with in vitro synthesized DENV gRNA (1X; as defined in the Section 2). BHK-21 cells were harvested at 24 or 48 h.p.t. with the DENV gRNA, and total RNA and proteins were extracted. The concentration of DENV gRNA was determined by RT-qPCR and expressed relative to the [DENV RNA] present at 24 h.p.t. The results show that DENV RNA increases by 54% at 48 h.p.t. of the DENV gRNA (Figure 2B). Confirming viral replication DENV non-structural protein 3 (NS3) could be readily detected by immunofluorescence in BHK-21 cells at 48 h post-DENV gRNA transfection (Figure 2C). Luciferase activities were measured and expressed as relative RLuc or FLuc activity, with the RLuc and FLuc levels from cells transfected with the dl DENV IRES plasmid alone set to 1 (Figure 2D–I). At 24 h.p.t., RLuc activity did not vary for the dl-plasmids (Figure 2D). As expected, FLuc activity was not detected in BHK-21 cells transfected with the dl ∆EMCV plasmid (Figure 2E). In the presence of the DENV gRNA, FLuc activity from the dl DENV IRES RNA increased by 63% (Figure 2E). An analysis of the FLuc/RLuc ratio (relative translational activity, RTA), as an index of IRES activity, with the RTA value obtained with the dl DENV IRES RNA alone set to 1, showed that at 24 h.p.t. of the DENV gRNA, no difference in DENV IRES activity existed (Figure 2F). At 48 h.p.t. of the DENV gRNA, RLuc of the dl DENV IRES RNA increased by 22%, while the RLuc activity of the dl ∆EMCV did not (Figure 2G). FLuc activity was not detected in cells transfected with the dl ∆EMCV plasmid, while FLuc activity from the dl DENV IRES RNA increased by 55% in cells replicating DENV (Figure 2H). An analysis of the RTA at 48 h.p.t. of the DENV gRNA (1X) showed a 26% increase in DENV IRES activity (Figure 2I). Thus, the results suggest that DENV IRES activity is stimulated in cells replicating DENV.

As an additional control, BHK-21 cells were transfected with the dl DENV IRES or with the dl ∆EMCV DNA (Figure 2A), and four h.p.t. cells were transfected, or not, with in vitro synthesized DENV gRNA (5X). BHK-21 cells were harvested at 24 h.p.t. with the DENV gRNA, and total RNA and proteins were extracted. The DENV gRNA was detected at 24 h.p.t (Figure 2J). Luciferase activities were measured and expressed relative to luciferase levels in cells transfected with the dl DENV IRES plasmid set to 1 (Figure 2K,L). At 24 h.p.t., RLuc activity was reduced in cells transfected with the DENV gRNA (Figure 2K), indicating that the impact of DENV replication on cap-dependent translation initiation is relative to the viral concentration (1X in Figure 2D–F vs. 5X in Figure 2K–M). Despite the reduction in cap-dependent translation initiation (Figure 2K), in the presence of the DENV gRNA (5X), FLuc activity from the DENV IRES RNA significantly increased by 41% (Figure 2L). Analysis of the RTA, relative to the dl DENV IRES RNA alone set to 1, showed that at 24 h.p.t. of the DENV gRNA (5X), DENV IRES activity increased by 61% (Figure 2M). Thus, the results show that even though cap-dependent translation is reduced (Figure 2K and [38]), DENV IRES is stimulated in cells replicating DENV.

### 3.3. DENV IRES Stimulation Induced by Human Rhinovirus 2A Protease Does Not Require Total Inhibition of Cap-Dependent Translation Initiation

**Figure 3:**
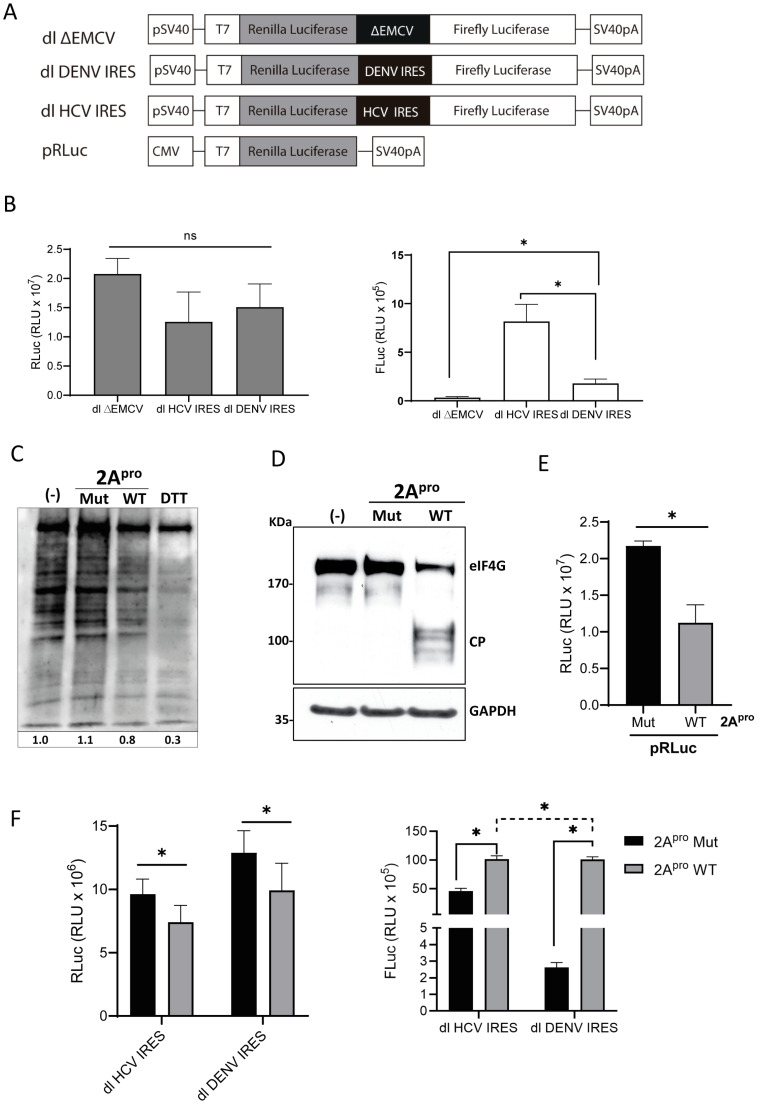
**DENV IRES activity is stimulated in BHK-21 cells expressing the HRV 2A protease.** (**A**) Schematic representation of the vectors used in the assay. (**B**) BHK-21 cells were transfected with the dl ∆EMCV, dl HCV IRES, or dl DENV IRES plasmids (200 ng). The activities of the RLuc (gray bars) and FLuc (white bars) were determined and expressed as RLU. (**C**–**F**) Plasmids pRLuc, dl HCV IRES, or dl 5′UTR DENV (200 ng) were transfected in BHK-21 cells together with a plasmid expressing the wild-type HRV-2A protease (p2A-WT) or an inactive mutant (p2A-Mut) (500 ng). (**C**) The impact of HRV p2A-WT or an inactive mutant (HRV p2A-Mut) on global protein synthesis was monitored by a SUnSET experiment [33,34]. The relative amount of puromycin-labeled protein was estimated by image-J. (**D**) The cleavage of eIF4G was monitored by Western blotting using polyclonal antibodies against eIF4G [35]. (**E**) RLuc activity of the pRLUc in the presence of HRV-p2A WT or HRV-p2A Mut was determined and expressed as RLU. (**F**) RLuc and FLuc activities from the dl-plasmids were measured and expressed as RLU. Statistical Analysis was performed using a *t*-test for RLuc analysis and a one-way ANOVA followed by a Tukey’s multiple comparison test for Fluc comparisons. Values represent the mean (±SEM) for three independent experiments, each conducted in duplicate (* *p* < 0.05, and ns, not significant).

To gauge the DENV IRES activity in BHK-21 cells, we compared it with that of the hepatitis C (HCV) IRES. The HCV IRES was selected for these experiments as it functions efficiently in BHK-21 cells [39]. So, BHK-21 cells were transfected with the dl ∆EMCV, dl HCV IRES, or the dl DENV IRES (Figure 3A). The dl HCV IRES harbors the 5′UTR of HCV 1b mRNA, the region that exhibits the IRES function, in the intercistronic space [18]. Luciferase activities were measured 24 h.p.t. and expressed as relative luciferase units (RLUs). The results showed that RLuc was within the same order of magnitude for all dl-plasmids (Figure 3B). However, in agreement with our previous report [7], the FLuc expression from the DENV 5′UTR was significantly lower than that of the HCV IRES (Figure 3B). However, despite the considerably weaker activity of the DENV IRES in BHK-21, the FLuc activity from the dl DENV IRES was statistically higher than that of the dl ∆EMCV negative control.

The presence of L^pro^ stimulates DENV-like RNA cap-independent translation initiation (Figure 1). An earlier report showed that DENV IRES activity is stimulated in cells expressing the 2A^pro^ [7]. Thus, inhibiting cap-dependent translation initiation by L^pro^ and 2A^pro^ expression is expected to stimulate DENV IRES activity. If so, it would be reasonable to predict that the enhancement of DENV IRES activity would be proportional to the shutdown of cap-dependent translation initiation. However, the earlier report did not evaluate this possibility [7]. Knowing that 2A^pro^ is less efficient and has slower kinetics in cleaving eIF4G than L^pro^ [36,40,41] and that the percentage of cleavage of eIF4G by 2A^pro^ is concentration-dependent [42], the experimental condition needed to express sufficient 2A^pro^ in BHK-21 cells to obtain a partial protein synthesis shutdown was established. A plasmid expressing an inactive mutant protease (2A^pro^-Mut) was used as a negative control. A SUnSET experiment was conducted to confirm the partial reduction in global protein synthesis in the presence of de 2A^pro^-WT [33,34]. The results show that under the used experimental conditions, a 27% (2A^pro^-Mut vs. 2A^pro^-WT) reduction in global protein was observed (Figure 3C). Next, BHK-21 cells were transfected with a monocistronic vector encoding for RLuc (pRLuc), the dl-plasmids dl HCV IRES, or dl DENV IRES and the 2A^pro^-WT- or 2A^pro^-Mut-encoding DNA. Plasmid pRLuc was used as an independent control for cap-dependent translation initiation. The partial cleavage of eIF4G in the presence of 2A^pro^-WT was confirmed by a Western blot analysis (Figure 3D). As anticipated, no cleavage of eIF4G was evidenced in cells transfected with the 2A^pro^-Mut (Figure 3D). Transfection of BHK-21 cells with the 2A^pro^-WT encoding plasmid led to a 50% reduction in RLuc activity from the pRLuc RNA (Figure 3E), confirming a partial decrease in cap-dependent translation initiation under the used experimental conditions. Consistent with the SUnSET result (Figure 3C), the presence of the 2A^pro^-WT reduced RLuc activity by an average of 23% for either vector compared to its 2A^pro^-Mut control (Figure 3F). In the presence of the 2A^pro^-WT, FLuc activity from the HCV IRES increased by 120% compared to its 2A^pro^-Mut control (Figure 3F). Strikingly, FLuc activity from the dl DENV IRES significantly increased to levels comparable to those exhibited by the dl HCV IRES under similar treatment (Figure 3F). This observation confirms that treatment of cells with 2A^pro^-WT strongly enhances DENV IRES activity [7]. However, the inhibition of cap-dependent translation, associated with the cleavage of eIF4G, alone cannot explain the increase in HCV IRES and DENV IRES activity in BHK-21 cells. Furthermore, 2A^pro^ enzymatic activity is required to stimulate the DENV IRES.

### 3.4. PTB Knockdown Negatively Impacts DENV-IRES Activity

Activation of picornaviral IRESs induced by L^pro^ and 2A^pro^ has been associated with the cleavage of eIF4G (inhibition of cap-dependent translation initiation) and other cellular protein targets [43,44,45,46,47,48]. In addition, 2A^pro^ disrupts the nuclear–cytoplasmic transport of proteins, leading to an accumulation of nuclear–cytoplasmic shuttling proteins in the cytoplasm [44,45]. In the context of human rhinovirus (HRV) mRNA, most delocalized proteins act as ITAFs, stimulating the HRV IRES [45,49]. Therefore, one plausible explanation for the above results (Figure 3) is that DENV IRES activity requires an ITAF that enhances the HRV IRES activity. Among the known HRV IRES ITAFs [49], we decided to study the impact of PTB, an abundant and ubiquitously expressed protein [50], on DENV IRES activity.

PTB was considered an ideal candidate ITAF for the DENV IRES because protein abundance increases at the early stages of DENV infection (12 h.p.i) in Vero cells [51], and PTB binds the DENV vRNA during infection and is known to play a role in DENV replication [51,52,53,54,55]. Also, one report showed that PTB overexpression was enhanced, and its knockdown reduced DENV vRNA translation [52]. Thus, PTB modulates DENV protein synthesis [52]. PTB is also an ITAF for several viral IRESs, including the HRV IRES [50,56] and IRESs of other members of the *Flaviviridae*, such as HCV and Zika [25,57]. However, the specific role of PTB as an ITAF for the DENV IRES has not been evaluated.

**Figure 4:**
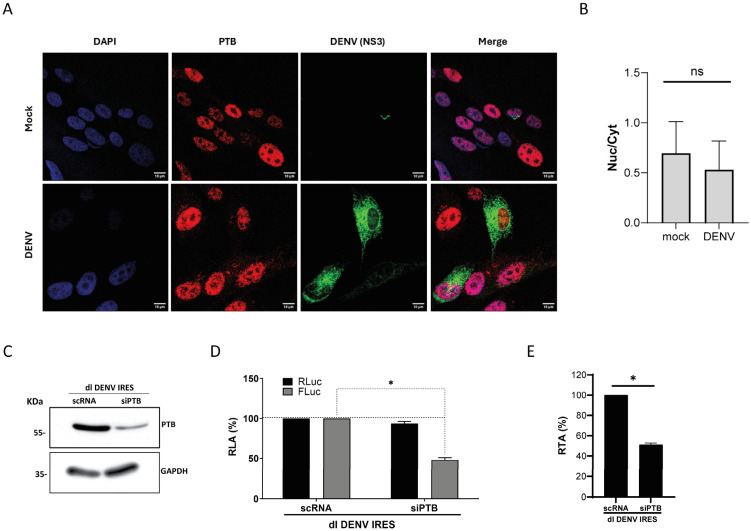
**Endogenous PTB localization in BHK-21 cells and impact of PTB knockdown on DENV IRES activity.** (**A**) BHK-21 cells were infected with DENV2 NGC or not (Mock). At 48 h.p.i., PTB and DENV were detected using an anti-PTB antibody or anti-DENV NS3 antibody, respectively. Vectashield with DAPI was used as a mounting medium. Images were captured and analyzed by confocal laser microscopy (Nikon C2+, Melville, NY, USA) and processed by Image J. Scale bar = 10 μm. (**B**) PTB’s nuclear and cytoplasmic distribution in DENV-infected (NS3 positive; DENV) and non-infected (mock) cells was roughly estimated by determining the ratio between nuclear and cytoplasmic mean fluorescence intensity (MFI). The values shown were obtained from three full images captured and analyzed by confocal laser microscopy, including the one used to extract panel (**A**). Statistical analysis was performed using a *t*-test (* *p* < 0.05). (**C**–**E**) BHK-21 cells were transfected with a scrambled RNA (sc) or siRNA designed to target PTB RNA (PTB isoforms and paralogs) and the dl DENV IRES plasmid. (**C**) Knockdown of PTB 24 h.p.t. was confirmed by Western blotting using an anti-PTB antibody. GAPDH was detected using an anti-GAPDH antibody (αGAPDH) as a loading control. (**D**,**E**) RLuc and FLuc activities were measured 24 h.p.t. and expressed relative to the scRNA set to 100% as RLA (**D**) or RTA (**E**). The values shown are the mean (+/− SEM) of three independent experiments, each conducted in duplicate. Statistical analysis was performed using a *t*-test (* *p* < 0.05).

DENV replication induces PTB relocalization from the nucleus to the cytoplasm in Vero cells but not in HuH-7 cells [52,54]. To understand what occurred in BHK-21 cells, we infected them with DENV-2, as described in the Section 2. Viral replication was monitored by following the expression of the DENV NS3 protein. In uninfected BHK-21 cells, we found that PTB is mainly localized in the cell nucleus (Figure 4A,B). However, in some BHK-21 cells actively replicating DENV, PTB was partially translocated to the cytoplasm (Figure 4A,B).

Even though PTB was mainly localized in the cell nucleus in DENV-replicating BHK-21 cells (Figure 4A,B), we wondered if PTB knockdown impacted DENV IRES activity. For this, BHK-21 cells were transfected with the dl DENV IRES vector and a cocktail of short interfering RNAs (siPTBs), targeting the mRNAs of endogenous PTB isoforms (PTB1, PTB2, and PTB4) and paralog (nPTB), or with a non-related scrambled control siRNA (scRNA), as described in [17,25]. The PTB paralog nPTB was also targeted because its levels increase when PTB isoforms are knocked down [54]. In addition, nPTB is also an ITAF for other IRESs [58]. The reduction in endogenous PTB by siPTB treatment was confirmed by a Western blot analysis (Figure 4C). Luciferase activities were then measured and expressed as relative luciferase activity (RLA), with the RLuc and FLuc levels from cells transfected with the scRNA set to 100% (Figure 4D). No change in RLuc activity was observed, while a significant decrease (48%) of FLuc was observed in cells transfected with the dl DENV IRES plasmid and treated with the siPTB (Figure 4D). Analysis of the RTA confirmed the significant reduction in DENV IRES activity in cells treated with siPTB (49%) (Figure 4E). These results suggest that PTB plays a role in DENV IRES-mediated translation initiation, without having an impact on cap-dependent translation initiation.

### 3.5. The Overexpression of PTB Isoforms Stimulates DENV IRES Activity

**Figure 5:**
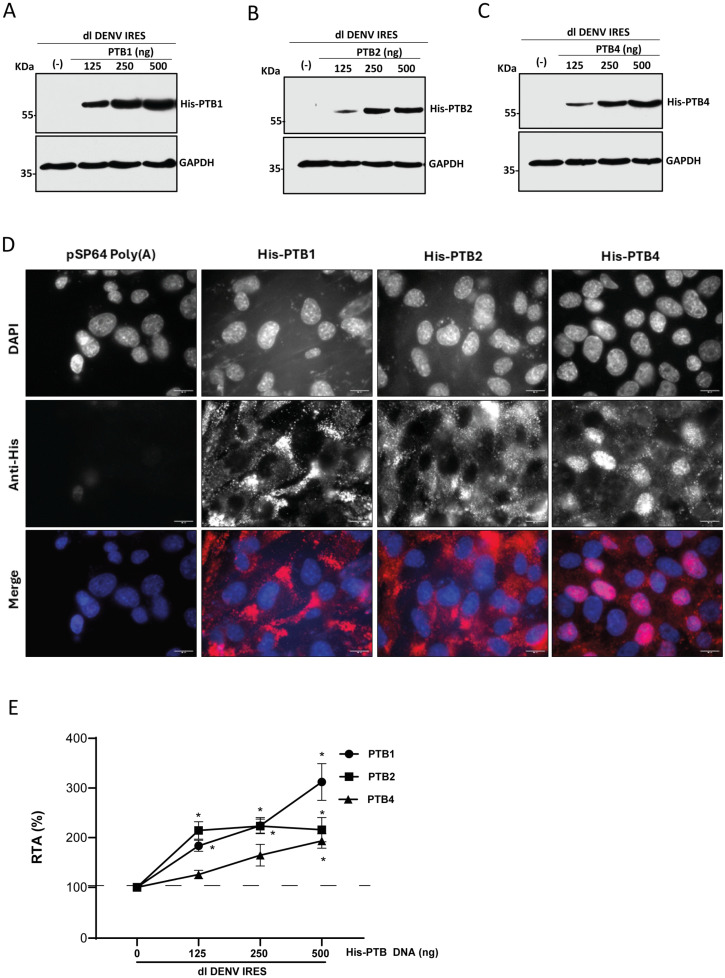
**PTB1, PTB2, and PTB4 stimulate DENV IRES activity in BHK-21 cells.** (**A**–**D**) BHK-21 cells were transfected with the dl DENV IRES plasmid (200 ng) and different concentrations (125, 250, or 500 ng) of a vector expressing a His-tagged version of PTB1, PTB2, or PTB4. Cells transfected with the pSP64 Poly(A) vector were used as the negative control (−). Expression of the tagged proteins, PTB1 (**A**), PTB2 (**B**), and PTB4 (**C**), was confirmed by Western blotting using an anti-His antibody (αHis). GAPDH was detected using an anti-GAPDH antibody (αGAPDH) as a loading control. (**D**) In parallel, at 24 h.p.t, the expression of His-PTBs was detected by IF using an anti-His antibody in BHK-21 cells transfected with the dl DENV IRES plasmid (200 ng) and a vector expressing a His-tagged PTB1, PTB2, PTB4 (500 ng), or pSP64 Poly(A). Vectashield with DAPI was used as a mounting medium. Images were captured and analyzed by fluorescence microscopy (Olympus BX51 Microscope) and processed by Image J. Scale bar = 10 μm. (**E**) IRES activity was measured and expressed as RTA (%) relative to the activity measured in the BHK-21 cells transfected with the pSP64 Poly(A)vector. Values are the means (+/− SEM) from three independent experiments. Statistical analysis was undertaken by the ANOVA test followed by Dunnett’s multiple comparisons test, * *p* < 0.05 v/s the negative control.

Due to alternative splicing, three isoforms of PTB (PTB1, PTB2, and PTB4) are generated in cells [50]. Interestingly, the different PTB isoforms impact mRNA splicing and translation differentially [17,25,59]. Given that PTB knockdown negatively impacted DENV IRES activity (Figure 4), we explored whether PTB1, PTB2, and PTB4 played a role in DENV IRES-mediated translation initiation. Thus, BHK-21 cells were transfected with dl DENV IRES vector and plasmids encoding for His-PTB1, His-PTB2, His-PTB4, or the control DNA pSP64 Poly(A). At twenty-four h.p.t., protein lysates were obtained, and the overexpression of His-PTB1 (Figure 5A), His-PTB2 (Figure 5B), or His-PTB4 (Figure 5C) was confirmed by Western blotting using an anti-His antibody and detecting GAPDH as a loading control. Subcellular localization of the overexpressed His-PTB isoforms in cells was followed in a parallel experiment by an IF assay (Figure 5D). Overexpressed His-PTBs were identified in the cell’s nucleus and cytoplasm. However, for His-PTB4, a more nuclear signal was evident (Figure 5D). Luciferase activities were measured, and data were expressed as RTA, with the values from cells transfected with the dl DENV IRES plasmid and the control DNA (-) set to 100% (Figure 5E). The overexpression of His-PTB1, His-PTB2, and His-PTB4 stimulates DENV IRES activity. However, the stimulation of DENV IRES activity was not equivalent for the three PTB isoforms (Figure 5E). The stimulation hierarchy for the DENV IRES was PTB1 (200%) > PTB2 (100%) > PTB4 (93%).

### 3.6. RNA Recognition Motifs Participate in PTB1, PTB2, and PTB4 ITAF Activity on DENV IRES

**Figure 6:**
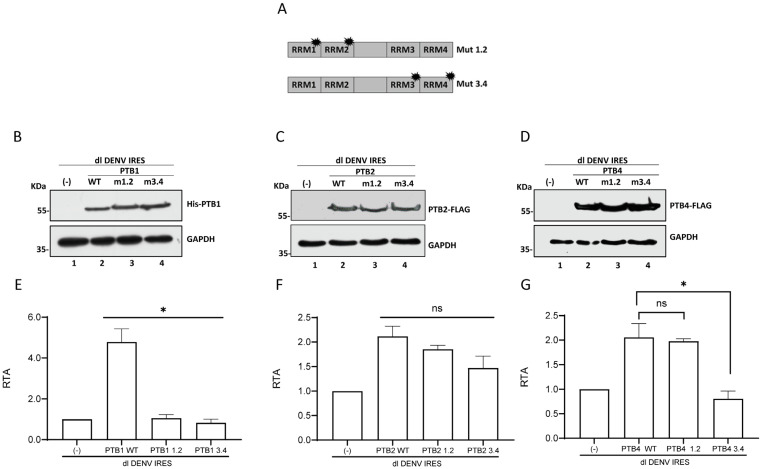
**PTB1, but not PTB2 or PTB4, requires RRM1/RRM2 and RRM3/RRM4 to stimulate the DENV IRES activity in BHK-21 cells**. (**A**) Schematic representation of PTB proteins showing the mutant RRMs [17,25]. (**B**–**G**) BHK-21 cells were transfected with the dl DENV IRES plasmid (200 ng) and a vector expressing His-tagged versions of PTB1 (WT), PTB1mut1.2 or, PTB1mut3.4 or a FLAG-tagged version of PTB2 (WT), PTB2mut1.2 or, PTB2mut3.4 and FLAG-tagged version of PTB4 (WT), PTB4mut1.2 or, PTB4mut3.4. Cells were transfected with the pSP64 Poly(A) vector as a control (-). Expression of the tagged proteins was confirmed by Western blotting using an anti-His (αHis) (B) or anti-FLAG (αFLAG) (**C**,**D**) antibody. GAPDH was detected using an anti-GAPDH antibody (αGAPDH) as a loading control. IRES activity was measured and expressed as RTA (%) relative to the activity measured in the BHK-21 cells transfected with the pSP64 Poly(A). Results for PTB1 and its mutants are shown in (**E**); for PTB2, in (**F**); and for PTB4 and its mutants, in (**G**). Values are the means (+/− SEM) from three independent experiments. Statistical analysis was performed by using an ANOVA test followed by Dunnett’s multiple comparisons tests (* *p* < 0.05, and ns, not significant).

PTB binds the DENV vRNA [53,54,55], and its interaction with the 3′UTR of the DENV vRNA has been characterized [52,60]. However, no PTB binding site in the 5′UTR of the DENV vRNA has been reported. Nonetheless, we sought to evaluate the impact of mutating PTB RNA recognition motifs (RRMs) on DENV IRES activity. PTBs have four RRMs (RRM1–RRM4) [61]. Compared to PTB1, PTB2 and PTB4 contain 19 and 26 additional amino acids between RRM2 and RRM3, respectively [50,59]. So, the linker length between RRM2 and RRM3 varies among the PTB isoforms. Therefore, to determine the putative role of the RRMs on the ITAF activity of PTB1, PTB2, and PTB4, the previously reported PTB1 and PTB4 mutants, PTBmut1.2 and PTBmut3.4, were used [17,19]. Also, the PTB2-FLAG mutants PTB2mut1.2 and PTB2mut3.4 were generated as indicated in the Section 2 (Figure 6A). Mutations in the different RRM domains correspond to the m (RRM1), b (RRM2), f (RRM3), and k (RRM4) mutants shown to disrupt the respective RRM’s ability to bind RNA [62]. The rationale for developing the mutant RRM1/RRM2 and RRM3/RRM4 as clusters considered that PTB1, PTB2, and PTB4 defer only in the linker domain between RRM2 and RRM3 and that RRM3 and RRM4 act as a coordinated pair [50,59,61,63].

Next, we evaluated the impact of PTBmut1.2 and PTBmut3.4 on DENV IRES activity. For this, the dl DENV IRES plasmid was cotransfected in BHK-21 cells with a control DNA (-), with His-PTB1, His-PTB1mut 1.2, His-PTB1mut3.4, PTB2-FLAG, PTB2mut 1.2-FLAG, PTB2mut3.4-FLAG, PTB4-FLAG, PTB4mut 1.2-FLAG, or the PTB4mut3.4-FLAG. The presence of the recombinant proteins in transfected BHK-21 cells was confirmed by Western blotting using an anti-His antibody for PTB1 (Figure 6B) or an anti-FLAG antibody for PTB2 (Figure 6C) and PTB4 (Figure 6D). The expression of His-PTB1 (Figure 6E) stimulated DENV IRES activity to a greater extent than PTB2-FLAG or PTB4-FLAG (Figure 6F,G). Stimulation of DENV IRES activity by PTB2-FLAG and PTB4-FLAG was similar. Inactivation of either RRM1/RRM2 or RRM3/RRM4 abrogated stimulation of the activity of the DENV IRES by His-PTB1 (Figure 6E). The inactivation of RRM1/RRM2 or RRM3/RRM4 in the context of PTB2-FLAG had no impact on the ability of the protein to stimulate the DENV IRES (Figure 6F). In PTB4-FLAG, mutant RRM3/RRM4, but not RRM1/RRM2, abrogated the stimulation of DENV IRES activity (Figure 6G). Thus, the results show that in the case of PTB1, both domains RRM1/RRM2 and RRM3/RRM4 are necessary to stimulate DENV IRES activity (Figure 6E), whereas, for PTB4, only RRM1/RRM2 is sufficient to enhance IRES activity (Figure 6G). In the case of PTB2, only one RRM pair is necessary to ensure the stimulation of the DENV IRES (Figure 6F). Together, the results suggest that PTB ITAF function is associated with its ability to bind RNA.

## 4. Discussion

**Figure 7:**
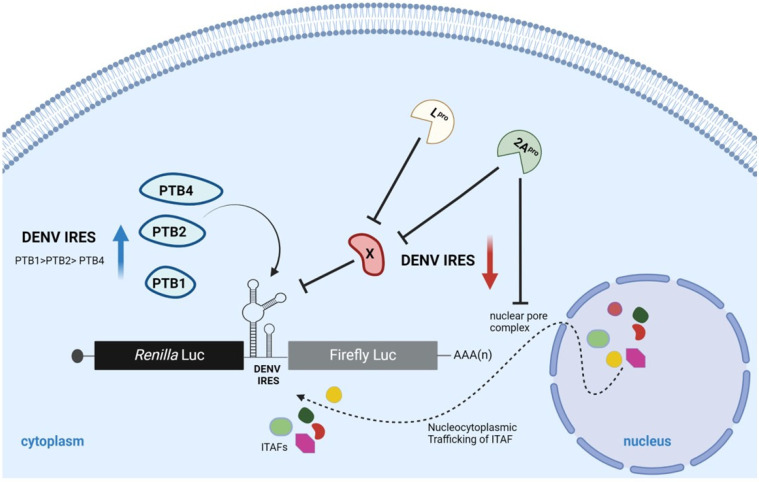
**PTB stimulates DENV IRES-mediated translation initiation.** The proposed model predicts that PTB1, PTB2, and PTB4 bind the DENV RNA, acting as stimulatory ITAFs for the DENV IRES. Furthermore, the results generated using the picornavirus L^pro^ and 2A^pro^ suggest that besides eIF4G cleavage [74,75], an inhibitory protein (X) that represses DENV IRES activity in BHK-21 cells is targeted by L^pro^ and 2A^pro^. The cleavage of X by L^pro^ and 2A^pro^ would derepress DENV IRES activity. Alternatively, 2A^pro^ may disrupt nuclear–cytoplasmic protein transport, accumulating nuclear proteins in the cytoplasm [44,45,70]. In the cytoplasm, the relocalized nuclear proteins could act as ITAFs stimulating DENV IRES activity. The scheme was created by L. Feranandez-Garcia using BioRender (Fernandez, L. (2024) BioRender.com/r99n554).

DENV vRNA translation initiation can transition from canonical cap-dependent to a cap-independent mode when cap-dependent translation is hindered [5,38,64]. The molecular mechanisms driving the vRNA transition from cap-dependent to cap-independent translation initiation remain unclear. Furthermore, two very different mechanisms could explain how the DENV vRNA translation thrives when cap-dependent translation initiation is shut off [5,6]. The first corresponds to a 5′-3′end-dependent-IRES-independent mechanism [5], and the second is an IRES-mediated initiation mechanism [6,7]. In this study, using monocistronic mRNAs, we confirm that DENV cap-independent translation initiation is functional, albeit to low levels in BHK-21 cells (Figure 1) [7]. In cells, the 5′cap is recognized by eIF4E, the cap-binding protein from the eIF4F complex [65,66]. The 5′cap (m^7^GpppN) recognition by eIF4E is pivotal in cap-dependent translation initiation [65,66]. However, eIF4E does not recognize the 5′Acap (ApppA) [67]. Nonetheless, cap-independent translation initiation, which is independent of the mRNAs 5′end, should not be affected by a 5′Acap. Consistent with earlier reports [4,7], replacing an active 5′cap with an inactive 5′Acap (ApppA) in the context of a monocistronic DENV-like mRNA supported but did not stimulate cap-independent translation in BHK-21 cells (Figure 1). This result indicates that the low activity of DENV cap-independent translation initiation in BHK-21 cells is not associated with an active *cis*-competition between cap-dependent and cap-independent mechanisms for components of the translational machinery. This finding seems to directly contradict the observation that cap-independent translation from the monocistronic virus-like DENV-FLuc RNA was significantly stimulated in L^pro^ or 2A^pro^ expressing BHK-21 (Figure 1E) or HEK293T cells [7]. Findings using the L^pro^ indicate that, like for other viral IRESs, the cleavage product of eIF4G can support DENV IRES-mediated translation initiation [7,37]. Hence, our results confirm that the activity of the DENV IRES is eIF4E-independent. Even though this observation is relevant for dissecting the molecular mechanism that drives DENV IRES activity, it is not necessarily associated with the replication cycle of DENV, as the assembly of the eIF4F complex is not impaired during DENV infection [38]. Additionally, and independent from eIF4G cleavage, L^pro^ or 2A^pro^ may exert their function of enhancing DENV cap-independent translation initiation through the cleavage of an unknown target. Further validating this possibility, an enhancement of DENV IRES activity was evidenced when the 2A^pro^ was used in such a way that eIF4G cleavage was incomplete, and cap-dependent translation initiation was not fully inhibited (Figure 3). Total shut off of cap-dependent translation initiation associated with eIF4G cleavage was not an absolute requirement to stimulate DENV IRES activity in BHK-21 cells (Figure 3). These results might suggest that besides eIF4G, the 2A^pro^ targets additional host protein(s) that impact DENV IRES activity. One possibility is that 2A^pro^ targets a protein that hinders DENV IRES-mediated translation initiation in BHK-21 cells (protein X in Figure 7). This mechanism of IRES regulation by an inhibitory protein is not unprecedented; for example, Gemin5 is an RNA-binding protein (RBP) interacting with the 5′UTR of the FMDV vRNA, down-regulating IRES-mediated translation initiation [68,69]. Cleavage of Gemin5 by the L^pro^ relieves FMDV IRES-mediated translation initiation [43]. Interestingly, the repressor effect of Gemin5 on FMDV IRES-mediated translation initiation is associated with its ability to compete with PTB for binding to the FMDV vRNA [69]. The binding of either protein to the FMDV vRNA would induce a local RNA structural reorganization, determining RNA structures that are translationally active (PTB) or not (Gemin5) [69]. Alternatively, it is well known that 2A^pro^ disrupts the nuclear–cytoplasmic transport by targeting proteins that are components of the nuclear pore complexes [44,45,70]. In this scenario, nuclear proteins defuse and accumulate in the cell cytoplasm, where many act as ITAFs for cellular and viral IRESs (Figure 7). Even though plausible and scientifically attractive, these mechanisms of action of the picornaviral proteases were not evaluated in the present study. Further experiments are required to fully characterize the mechanism(s) by which the L^pro^ (Figure 1) and 2A^pro^ (Figure 3) stimulate cap-independent translation initiation from the DENV-like vRNA or DENV IRES activity from the dl-vector.

PTB was selected as an ITAF candidate for the DENV IRES. Consistent with a role in DENV IRES-mediated translation initiation, the knockdown of endogenous PTBs reduced DENV IRES activity (Figure 4). These findings are consistent with a previous report that used DENV-replicon to show that silencing PTB reduces DENV vRNA translation [52]. Nonetheless, the role of PTB in DENV vRNA translation remains controversial, as another study using virus-like RNAs indicated that PTB does not participate in DENV vRNA translation [54]. In both studies, the used RNAs could initiate translation using a cap-dependent, 5′-3′end-dependent IRES-independent, or IRES-dependent mechanism [2,3,4,5,6,7]. How the different mechanisms of translation initiation of the DENV mRNA are regulated in different cell types and during the various stages of the virus replication cycle remains unknown. Therefore, the precise mechanism or combination of mechanisms of translation initiation used by the DENV-replicon or the virus-like RNAs in the previous reports is complex to anticipate [52,54]. In sharp contrast with the earlier reports [52,54], herein, we exclusively evaluated the role of PTB on DENV IRES activity by isolating the 5′UTR of the DENV vRNA and studying it in the context of a bicistronic mRNA (Figure 4 and Figure 5). When overexpressed in BHK-21 cells, PTB1, PTB2, and PTB4 are ITAFs that stimulate (PTB1 > PTB2 > PTB4) DENV IRES-mediated translation initiation (Figure 5; Figure 7). So, as reported for other IRESs [17,25,59], our results show that PTB isoforms differentially impact the function of the DENV IRES (Figure 5; Figure 7). The differential effect of PTB isoforms on DENV IRES activity could result from protein subcellular localization, as His-PTB1 and His-PTB2 concentrated in the cell cytoplasm, while His-PTB4 localizes mainly to the cell nucleus (Figure 5).

How PTB isoforms mediated DENV IRES stimulation remains unclear. However, experiments using the mutant PTBs suggest that DENV IRES stimulation requires PTB binding to the mRNA (Figure 6). The PTB RRM recognizes polypyrimidine tracts in the RNA target containing CU-rich elements [71,72]. RRM1 and RRM2 are independent domains connected by flexible linkers, whereas RRM3 and RRM4 have a fixed relative orientation [61]. Four RRMs enable PTBs to interact with their target RNAs in multiple sites and alternative orientations [61]. This RNA recognition ability of its RRMs grants PTBs the capacity to bridge two pyrimidine tracts within a single RNA molecule, bringing them into proximity [61,73]. Consistent with this observation, the mutation of RRM1/RRM2 or RRM3/RMM4, known to disrupt RRM-RNA interactions [62], abolished the capacity of PTB1 to stimulate DENV IRES (Figure 6), suggesting that PTB1-induced stimulation of DENV IRES activity is associated with the protein’s ability to simultaneously bind different RNA regions of the short 96 nt sequence of the 5′UTR of the DENV-2 RNA. The precise sites for PTB1 binding on the DENV 5′UTR remain unknown. Mutations in either RRM1/RRM2 or RRM3/RMM4 did not impact the ability of PTB2 to stimulate the DENV IRES (Figure 6), while only mutations in RRM3/RMM4 abrogated the stimulation of DENV IRES-mediated by PTB4 (Figure 6). These data suggest that the mechanism of action used by PTB2 or PTB4 does not seem to be through bridging two pyrimidine tracts within the RNA molecule. One alternative mechanism of action could be that PTB2 and PTB4 establish protein–RNA interactions and protein–protein interactions with other ITAFs, favoring the assembly of ribonucleoprotein (RNP) complexes that regulate IRES-mediated translation initiation. Even though it is an attractive mechanism, it remains highly speculative as no other ITAF for the DENV IRES has been identified. Thus, further experimental data are required for its validation.

In conclusion, showing that PTBs are ITAFs for the DENV IRES expands our knowledge of the interaction network between the DENV IRES and cellular factors, providing new insights into translational control during DENV replication.

## Data Availability

All data is included in the text.
